# Controlling motile disclinations in a thick nematogenic material with an electric field

**DOI:** 10.1038/s41598-018-19891-0

**Published:** 2018-02-06

**Authors:** Amit Kumar Bhattacharjee

**Affiliations:** 1 Asutosh College, University of Calcutta, Kolkata, 700026 India; 2 Centre for Condensed Matter Theory, Department of Physics, Indian Institute of Science, Bangalore, 560064 India

## Abstract

Manipulating topological disclination networks that arise in a symmetry-breaking phase transformation in widely varied systems including anisotropic materials can potentially lead to the design of novel materials like conductive microwires, self-assembled resonators, and active anisotropic matter. However, progress in this direction is hindered by a lack of control of the kinetics and microstructure due to inherent complexity arising from competing energy and topology. We have studied thermal and electrokinetic effects on disclinations in a three-dimensional nonabsorbing nematic material with a positive and negative sign of the dielectric anisotropy. The electric flux lines are highly nonuniform in uniaxial media after an electric field below the Fréedericksz threshold is switched on, and the kinetics of the disclination lines is slowed down. In biaxial media, depending on the sign of the dielectric anisotropy, apart from the slowing down of the disclination kinetics, a nonuniform electric field filters out disclinations of different topology by inducing a kinetic asymmetry. These results enhance the current understanding of forced disclination networks and establish the presented method, which we call fluctuating electronematics, as a potentially useful tool for designing materials with novel properties *in silico*.

## Introduction

Topological singularities such as points, lines and walls are ubiquitous in phases with broken symmetry. Canonical examples include dislocations in solids^1^, vortex lines and rings^2^ in superfluid ^3^*He* and ^4^*He*, Abrikosov vortex lines in superconductors^3^, vortex lines in Bose-Einstein condensates^4^, umbilic lines^5^ and *π* solitons^6^ (disclinations) in nematic liquid crystals (NLC) that provide a testing ground for theories of cosmology^2,7^, *λ* lines in cholesteric fluids^8^, Bloch and Néel lines in ferromagnets^9^, walls in lipid membranes^10^ and string networks in ecology^11^. NLC phases display rich birefringence under a polarizing microscope during phase ordering from a disordered state after a rapid quench in pressure or temperature, resulting in the formation of disclinations with integer and fractional topological charge. These singularities proliferate after nucleation and form contractile loops after intercommutation^12^. Unlike dislocations, disclinations possess intricate kinetics, microstructure, and equivalence with an electric charge. Strings are charge neutral with either topological charge ±1 or ±1/2 residing at the two segments or end points of the string to form topological dipoles. Higher multipoles and integer charged dipoles also nucleate within the charge neutral strings at the early stage of kinetics. Subsequently, these structures rupture into fractionally charged dipolar strings. Similar to electrodynamics, like topological charges repel and unlike charges attract and annihilate in pairs while monopoles are nonexistent to retain charge neutrality unless created by symmetry-breaking boundaries, an inclusion of impurity or external drive with a laser beam^13^.

**Figure 1:**
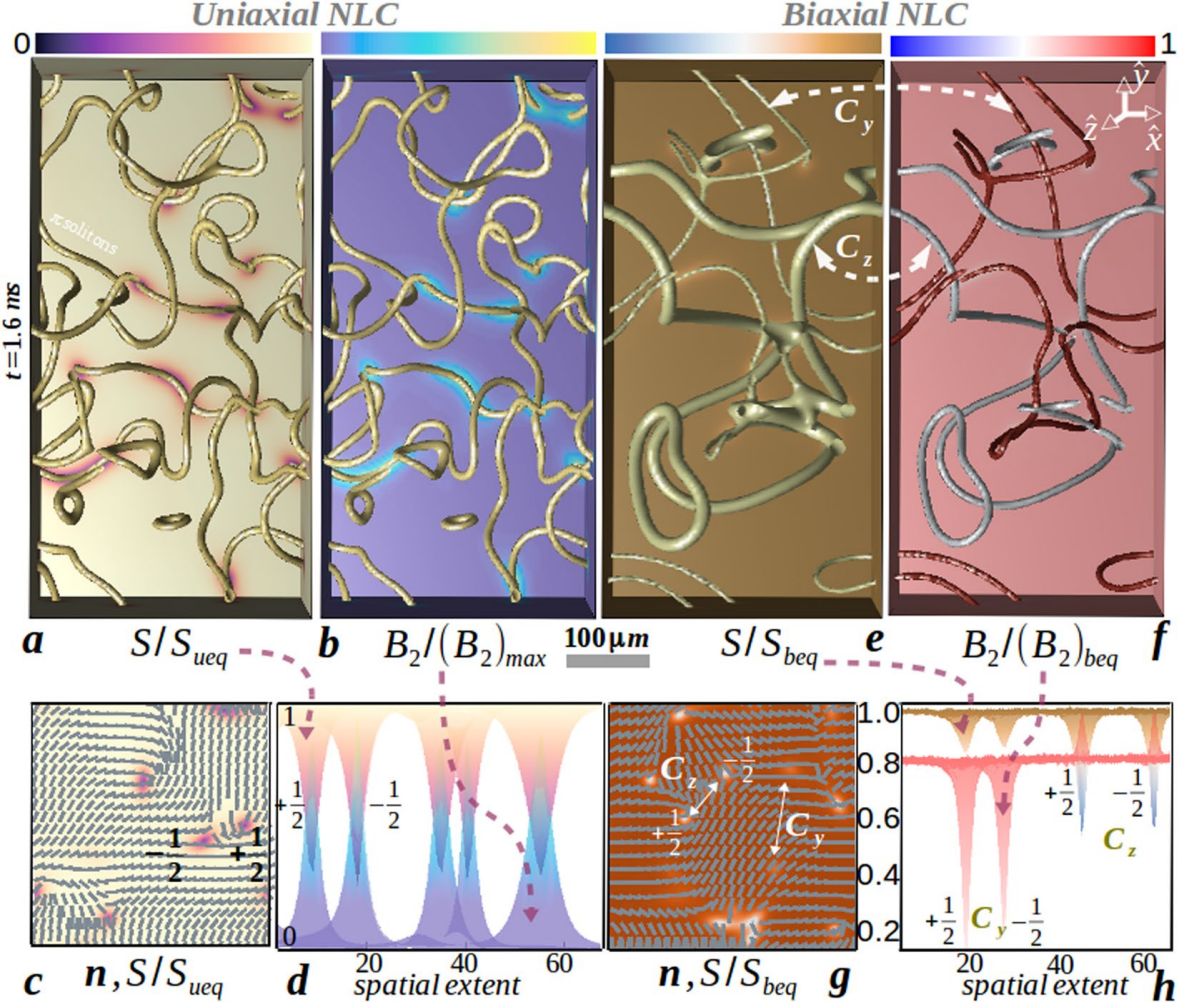
(**a**) *π* solitons in a thick uniaxial film of 5*CB* after a temperature quench from an isotropic state (*T* = 40 °C) to a nematic state (*T* = 33.65 °C). Supercooling and superheating temperatures are *T *= {34.2,34.47} °C. Disclination isosurfaces correspond to scalar uniaxial order with isovalue *S*_*ueq*_/2, where *S*_*ueq*_ = 0.086. (**b**) Corresponding biaxial order with isovalue (*B*_2_)_*max*_/2 where (*B*_2_)_*max*_ = 0.05. (**c**) uniaxial order and director distribution on a portion of the *xy*-slice plane of (**a**). (**d**) Corresponding spatial extension of scalar uniaxial and biaxial order, displaying core structure of two segments of charge neutral disclinations in-plane, which are ±1/2 integer defects^6,13^. (**e**) Charge neutral *π* solitons of different homotopy class in thermotropic biaxial media (see Supplementary Movie S2 and Methods for defect characterization scheme). Disclination isosurfaces correspond to isovalue 6*S*_*beq*_/7 where *S*_*beq*_ = 0.96. (**f**) Corresponding biaxial order with isovalues {0.42(*B*_2_)_*beq*_, 0.83(*B*_2_)_*beq*_} for homotopy class {*C*_*y*_, *C*_*z*_} where (*B*_2_)_*beq*_ = 1.2. (**g**) Uniaxial order and director distribution on a portion of the *xy*-slice plane of (**e**). Note the similarity in the microstructure of ±1/2 *C*_*z*_ defects with uniaxial defects in (**c**) and no variation in n for ±1/2 *C*_*y*_ defects. (**h**) The corresponding variation of uniaxial and biaxial order displaying core structure of disclination segment of both class in-plane. Although both scalar orders decrease in the core of *C*_*y*_ defect, biaxiality increases for a decreasing uniaxiality in *C*_*z*_ defect. Parameters are defined in Methods (section 3) and material (computation) parameters are tabulated in Table 1.

Existence, classification and recombination rules of disclinations in equilibrium, which play an important role in the material design, is governed by the energy landscape as well as the geometry (topology) of the order parameter space^12^. Strings in uniaxial NLC displayed in Fig. 1 (frames a–d) are topologically defined by ${\pi }_{1}( {\mathcal R} {{\mathscr{P}}}_{2})={{\mathbb{Z}}}_{2}$ with homotopy group *π*_1_ in the projective plane ${\mathcal R} {{\mathscr{P}}}_{2}$ resulting in the abelian group ${{\mathbb{Z}}}_{2}$ with topological charge ±1/2^12^. After a theoretical proposal^14^, *π* solitons have been seen in fluorescence confocal polarized-light microscopy of pentylcyanobiphenyl (5CB) NLC^6,7^, molecular simulations^15^ and field theoretic computations^13,16^. Likewise, biaxial disclinations displayed in Fig. 1 (frames e–h) are defined by ${\pi }_{1}( {\mathcal R} {{\mathscr{P}}}_{3})={{\mathbb{Q}}}_{8}$ where ${\mathcal R} {{\mathscr{P}}}_{3}$ is the projective plane and ${{\mathbb{Q}}}_{8}$ is the nonabelian group of quaternions generating three classes of half-integer topological charges denoted by *C*_*x*,*y*,*z*_. In monolayered thin films, the simultaneous and pairwise coexistence of fractionally charged point dipoles of either class *C*_*x*,*y*_, *C*_*y*,*z*_ or *C*_*x*,*z*_ is predicted^17^ and observed in field theoretic computations^16,18^. Albeit topologically proscribed in three dimensions, strings of disparate topology do not entangle but annihilate pairwise within the respective class^16^.

**Table 1:** Parameters values excercised to mimic uniaxial (upper row) and biaxial (lower row) thermotropic NLC.

Fig.	Γ(*P*^−1^1)	*A*(*Jcm*^−3^3)	*B*(*Jcm*^−3^3)	*C*(*Jcm*^−3^3)	*E*'(*Jcm*^−3^3)	*L*_1_(10^−7^7*dyn*)	*κ*	Θ	*ζ*(*μm*)	*k*_*B*_*T*(*J*)
1a–d 3	5					3.75 × 10^−3^	18	0.5	3.55	0
2a,b, 4	1	−8 × 10^−3^	−0.5	2.67	0	0.05	0	0		5 × 10^−6^
2c	5					0.05	0	0		
						6.8 × 10^−^^3^	9	0.5		
						3.75 × 10^−^^3^	18	0.5		
1e–h,7, 5,6	0.02	−4.5	−0.5	2.67	3.56	8.1	0	0	3.55	08 × 10^−3^

A rectangular simulation box of size 80^2^ × 160 *μm*^3^ with grid spacing Δ*x* = Δ*y* = Δ*z* = 1 *μm* and time step Δ*t* = 1*μs* is considered. We use material parameters for 5*CB* at *T* = 33.65 °C, *ε*_0_ = 1,*ε*_*a*_ = ±1, *ε*_*s*_ = 0.74 *ε*_*a*_^51^ and use earlier excercised material parameters for biaxial media^16,32^. Using equation (4), we estimate *E*_*F*_ = 2 × 10^−3^ *V*/*μm* for 5*CB* and with |*E*| = *E*_*F*_ × (10^−2^, 10^−1^,1), nondimensional parameters are *ε*_1_ = 1.5 × (10^−4^, 10^−3^, 10^−2^), *ε*_2_ = 1.8 × (10^−3^, 10^−2^, 10^−1^). In biaxial media, we use |*E*| = 1.5 *V*/*μm* < *E*_*F*_, *ε*_1_ = 0.14, *ε*_2_ = 3.35. *t*_*on*_ = 1 *ms* for both uniaxial and biaxial problem, while *t*_*off*_ = 5 *ms* for uniaxial and *t*_*off*_ = ∞ for biaxial problem. Parameters are defined in Methods (section 3).

Depending on the anisotropic elastic constants of the medium, soft disclinations are vulnerable to thermal fluctuations and external stimulus like an electromagnetic field. Regulated by the sign of the dielectric anisotropy constant of the material, an electric field at the Fréedericksz threshold can orient the nematic director along or perpendicular to the field direction. It is particularly interesting to examine whether locally uniform and nonuniform electric field can lead to a time-dilated kinetics of the disclination network^13^, and, how the anisotropy of the nematic orientation embedded in the dielectric tensor leads to nonuniformity in the local electric field. For example, nematic regions at the top of a colloidal inclusion are generated due to the asymmetric distribution of the field intensity^19^. Such control is hard to characterize in experiments, impossible in nanoscale molecular simulations and limited in field theoretic calculations due to numerical complexity, as ref.^19^. mentions, “molecular alignment in the inhomogeneous electric field has not yet been well studied as it is not easy to solve the Poisson equation with an inhomogeneous dielectric constant to calculate the local electric field”. Attributing to the scale invariant property of the Ginzburg-Landau-de Gennes (GLdG) field theory, relaxational kinetics of the orientation tensor has quantitatively reproduced experiments *in silico* from mesoscale^13,20^ to nanoscale^21^. *State of the art* grand challenge is attributed to the nonavailability of a robust numerical scheme^22^ which is, in descending order of complexity, (a) free from numerical artifacts of the traditional methods^23^, accounts for (b) local nonuniformity in electric field and (c) equilibrium thermal fluctuations by respecting physical laws, (d) guarantees zero-trace property of the orientation tensor and (e) incorporates anisotropic elasticity to probe beyond the single diffusion (one elastic constant) approximation^23^. Recent advances in fluctuating hydrodynamics of isotropic suspensions^24^ incorporating point (a) demand a natural, yet challenging, extension for anisotropic suspensions^25^ while on the other hand, numerical achievement of points (c–e) is fairly recent^20,26^.

By investigating beyond the uniform field assumption^27^, in this article, we have developed a fluctuating electronematics method based on the thermal description of the GLdG theory, with the physical control over the role of each forcing to accurately describe thermal and electrokinetic phenomena in three dimensional NLC media. Using simple analytical argument, we provide an understanding of the underlying mechanism responsible for the outcome in both uniaxial and biaxial media in the free draining limit at moderate to small electric field intensity, where the advective flow of the anisotropic media can be neglected. The interdependency between the topology in the orientational order of the media and morphology of the external field is elucidated through the measurement of disclination kinetics and morphology. It turns out that small magnitude of nonuniform electric field can significantly dilate the coarsening kinetics of disclination network and can eradicate certain topological class of disclinations in biaxial media.

## Results

### Thermoelectrokinetic effects of disclination network in a coarsening uniaxial NLC

Here we systematically probe on the role played by the elasticity of the medium and various external forcing (*k*_*B*_*T*, *E*) on the kinetics and microstructure of the string disclination assembly.

#### Role of thermal fluctuations and anisotropic elasticity

**Figure 2:**
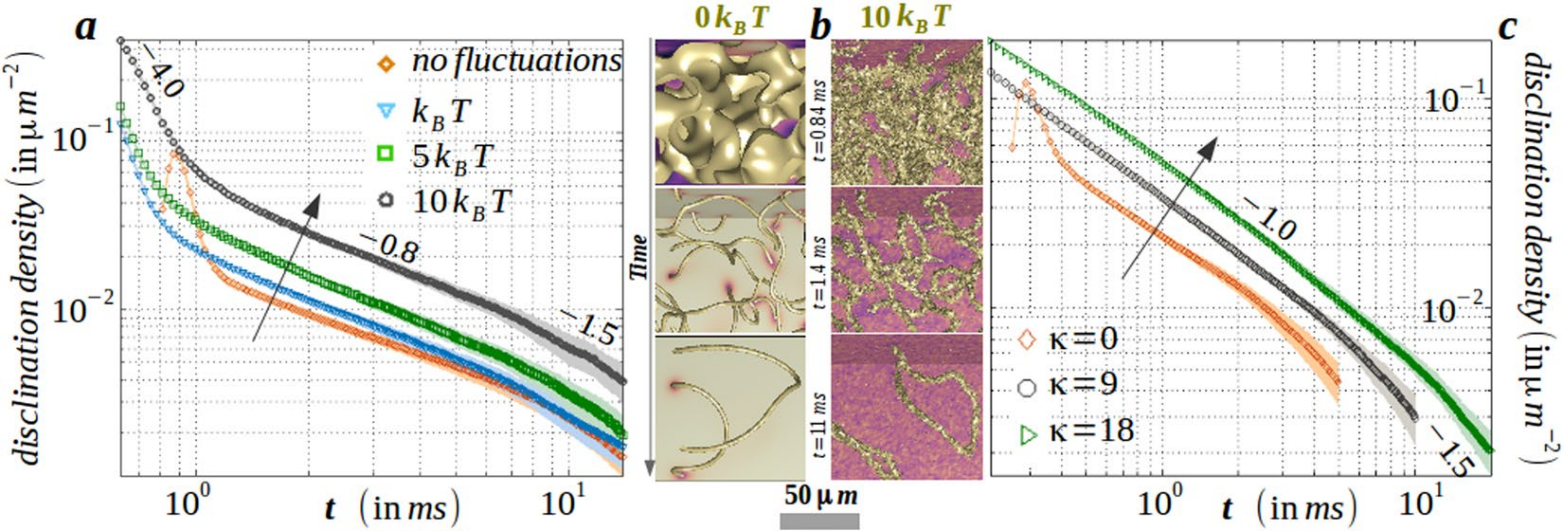
(**a**), Increment in disclination density with fluctuation amplitude. Note the three different slopes corresponding to initial diffusive regime → Porod’s law regime → late stage diffusive regime. (**b**) Comparison of disclination kinetics in a thermally fluctuating media with its athermal variant that corresponds to (**a**). A portion of the three-dimensional volume is shown for clarity, that displays domain decomposition before nucleation of disclinations → intercommuting disclinations → contractile disclination loops. (**c**) Increased elastic anisotropy leads to increased disclination density with a prolonged Porod’s law regime. Note that both thermal fluctuations and elastic anisotropy aids in early nucleation of the isotropic domain, but prolongs the disclination kinetics. The rendered colours in field values are indicated in Fig. 1 and the arrow denotes the increment direction. Material (computation) parameters are tabulated in Table 1.

The disclination kinetics is immensely influenced by external agents like thermal and electric forces, anisotropic elastic effects or shear. To illustrate the role of thermal fluctuations, we estimate the decay of disclination density per unit area in the degenerate elastic constant approximation without an electric field (*κ* = Θ = |*E*| = 0 in equation (7)). Using surface triangulation method^28^, we calculate the total surface area of the disclinations in the media. Figure 2: frame a plots the same for three different values of *k*_*B*_*T* including the athermal scenario and in frame (b) we show a portion of the three-dimensional volume that distinguishes the disclination kinetics between the athermal and thermal scenario. In the athermal scenario, three different regime with marked exponents emerges out in the evolution process, that had been previously quantified as diffusive regime → Porod’s law regime → diffusive regime^16^. The early diffusive regime corresponds to domain coarsening before nucleation of disclinations (*t* = 0.84 *ms*), while Porod’s law scale designates the defect annihilation kinetics (*t* = 1.4 *ms*) and finally, the late stage diffusion is attributed to contraction of isolated loops (*t* = 11 *ms*). Clearly, thermal fluctuations tend to increase the disclination surface density without affecting the scaling laws, that is important for materials (*e*.*g*. PAA) having transition temperature way above the room temperature. Fluid viscosity *η* can be obtained from the Stokes-Einstein relation *k*_*B*_*T*/*Kη* = constant. However, frame c shows that disclination density in the athermal media not only increases for an increase in *κ*, but also stretches the Porod’s law regime. The slope changes from 0.8 to 1 as elastic anisotropy is increased. While the slope of unity is also obtained in experiments with 5*CB*^7,29^, we re-establish our earlier claim^20^ about the crucial contribution of the anisotropic elasticity of the medium. Intuitively, anisotropic elastic constant results into asymmetric diffusion constants in Cartesian directions and thus brings asymmetry in the speed of ±1/2 integer point defects^23,30^. The loss of area in forming a contractile loop is greatly reduced for higher anisotropy.

**Figure 3:**
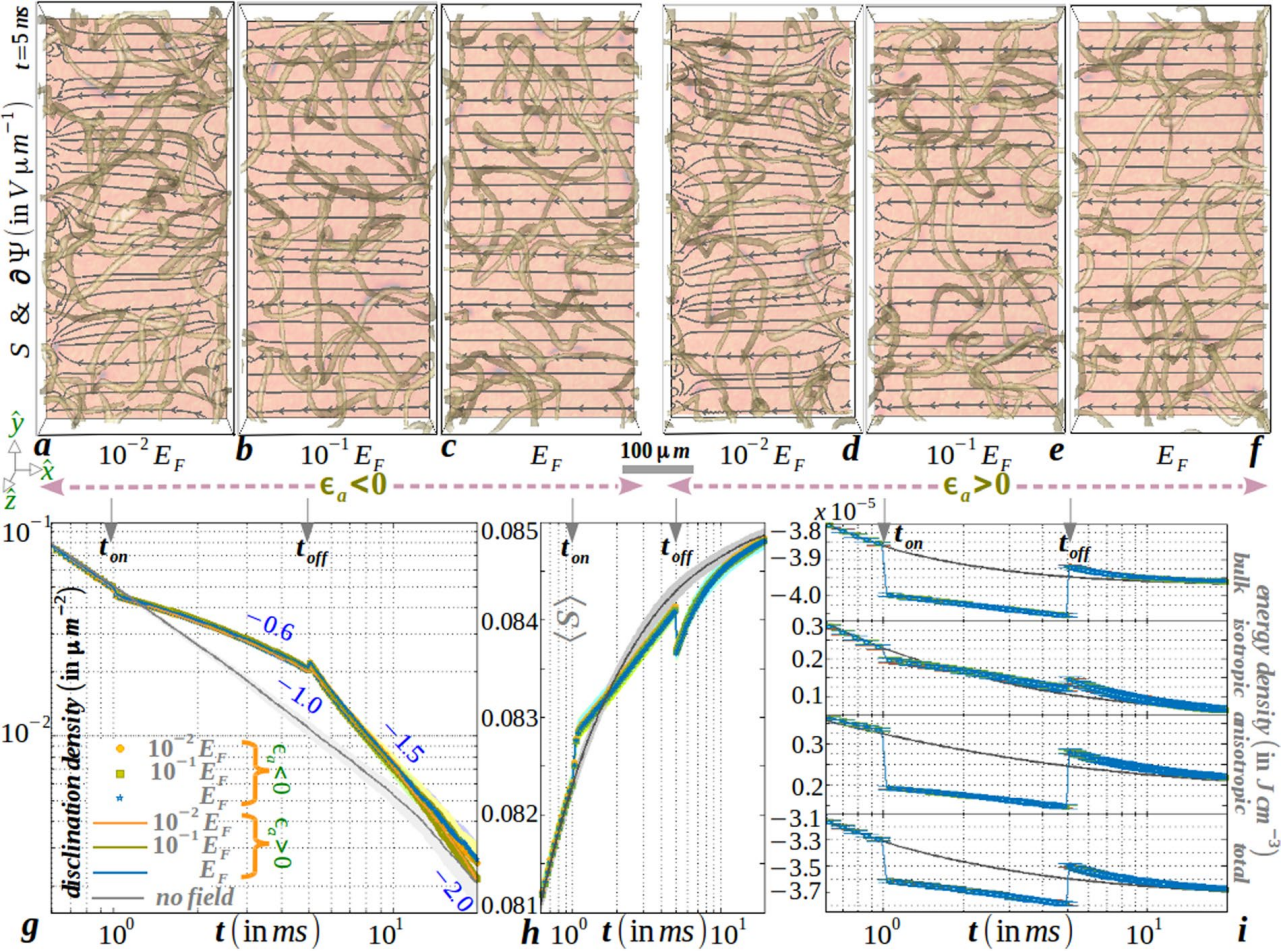
(**a**–**c**) Nonuniformity of electric flux lines along with the uniaxial order is sketched on slice plane *L*_*z*_/8 for negative dielectric anisotropy constant material (for example, butylmethoxybenzylidene (*MBBA*)) when the magnitude of the non-dimensionalized scalar potential is comparable to the orientation tensor (see Supplementary Movie S3). Disclinations in the bulk volume are also displayed to guide the eye. Uniformity in flux lines is gradually reached as the field is increased to the Fréedericksz threshold *E*_*F*_. (**d**–**f**) Comparatively similar electric response of a positive dielectric anisotropy constant material (for example, 5*CB*). (**g**) Evolution of disclination density per unit area displaying an increased life-span of *π* solitons with non-Markovian response during field cessation, (**h**) elastic response of the global uniaxial order and (**i**) (free) energy with varying field strength after onset and cessation of an unidirectional electric field. Equilibrium response is also sketched for comparison. Material (computation) parameters are tabulated in Table 1.

Before we embark on discussing coarsening in the presence of an electric field, we revisit the coarsening kinetics when no electric field is present. As seen in Supplementary Movie S1, disclinations with higher line tension and curvature are energetically disfavoured, culminating in stretched strings that form contractile loops after intercommuting with neighbouring strings. By taking into account the effect of viscous drag and length decrement of a string in forming a loop at time *t*, the disclination surface density *ρ* is found^7^ to scale as *ρ* ∝ *t* ^−1^. An identical scaling law is obtained for a planar disclination when equating the rate of change of the line tension per unit volume with the energy density loss rate^1^. Our method accurately reproduces the slope of 1 ± 10^−3^ within the errorbar shown in Fig. 3 (‘no field’ curve in frame g) with the material parameters of 5*CB*. Thermal fluctuations and elastic anisotropy lead to an increment in the disclination surface density at a given time and the period of disclination annihilation kinetics is extended without affecting the physical laws.

#### Role of an electric field

Next, we elaborate on the thermal and electrokinetic effects on coarsening in uniaxial NLC after the onset and cessation of an unidirectional voltage pulse. The electric field is applied at an instant *t*_*on*_ when only disclinations with dipolar charge ±1/2 are present, and, the medium is free of ±1 dipolar strings or point charges. The switch-off time of the field is set at an instant *t*_*off*_ when the electric flux lines within the medium do not change substantially. For a shallow quench below the supercooling line, for both signs of the dielectric anisotropy constant and below the Fréedericksz threshold of the electric field, the orientational order is comparable in magnitude with the non-dimensionalized electric potential. Thus within the medium, the flux lines are very much distorted as seen in Fig. 3. Instead of being aligned along the field direction, the isotropic cores of the strings displaying reduced uniaxial order are little deformed by the electric force^6^ and have a little contribution in distorting the flux lines. This is attributed to the weak coupling of scalar order to the electric field, unlike the director that strongly couples to the electric field. In no switch-off scenario (*t*_*off*_ → ∞), the nonuniformity of the flux lines is retained in the electrically forced nematic phase devoid of disclinations around *t*∼35 *ms* (not shown). The flux lines in Fig. 3 (frames a–f), however, tend to gain uniformity as the electric field is increased towards the Fréedericksz threshold for both signs of the dielectric anisotropy constant. As observed in frame g, electrically forced strings are little thinner due to a reduction of the surface density, but they are long lived due to a dilated kinetics. After the field is switched off, disclination kinetics and surface density do not immediately return to the zero-field behavior, but lag for an interval of $\sim 10\,ms$. Thus, the material retains a memory of the field onset in the process of exhibiting an elastic response. The electric field agitates the isotropic background towards an uniaxial medium, thus increasing the uniaxial order (frame h) and non-monotonically decreasing the total free energy (frame i). Though we obtain Brochard-Legér lines connecting *π* solitons under an intense field above the Fréedericksz threshold, the lack of backflow in our method cannot reproduce a ceasing motility of disclinations^6^. Rather, they annihilate at a faster rate due to the uncompensated electric drag force. Electrokinetic effects under intense forcing, such as electroconvection or rheochaos, can be quantitatively captured only if backflow is systematically included. Unlike in colloidal suspensions^24^, the question of the existence of correlations between fluctuations in orientation and velocity has to be answered from experiments^31^ before attempting a numerical study in which backflow effects are included.

We qualitatively argue about the slowing down of disclination kinetics for a planar isolated disclination loop. An in-plane estimate is valid for 5*CB* while twist constant is much smaller than splay or bend constants^20^. Also deep within the uniaxial phase where the director is aligned uniformly, Frank constant *K* can sufficiently define the medium elasticity^26^. In such situations, planar disclination energy per unit length is ${ {\mathcal F} }_{discl}=$ ${\int }_{{\mathbb{S}}}{d}^{2}x[K{({\boldsymbol{\partial }}f)}^{2}-{\varepsilon }_{0}{\varepsilon }_{a}\,{E}^{2}si{n}^{2}f\mathrm{]/2}$ where *f* = *kϕ* + *c*, with 0 ≤ *ϕ* ≤ 2*π*, *k* = ±1/2 being the topological charge, ${\mathbb{S}}$ the bounding plane and *c* a constant, defines a planar defect configuration *n* = [*cosf*, *sinf*]. Performing the surface integral, the reduced disclination energy per unit length is

1 $${ {\mathcal F} }_{discl}=\pi K{k}^{2}ln(\frac{\xi }{\zeta })-\frac{\pi {\varepsilon }_{0}{\varepsilon }_{a}}{4k}{E}^{2}({\xi }^{2}-{\zeta }^{2})+{ {\mathcal F} }_{discl}^{c},$$

where ${ {\mathcal F} }_{discl}^{c}$ is the disclination core energy. Equating the elastic energy ${ {\mathcal F} }_{discl}/\xi$ per unit area with the drag force −*η*∂_*t*_*ξ* per unit length yields contributions from (i) the equilibrium kinetics $\xi \sim {t}^{-\mathrm{1/2}}$ and (ii) the electric field effect $\xi \sim {e}^{\nu t}$ with *ν* = *πε*_0_*ε*_*a*_*E*^2^/8*kη*. While *ν* is independent of the sign of *k* or *ε*_*a*_, *ν* > 0 implies of a temporal reduction of the loop extinction kinetics. Physically this can be interpreted as a reduction in speed of approach between ±1/2-charged topological dipole within a charge-neutral loop due to the external forcing. As observed in Fig. 3 (frame g) and in supplementary Figure S1, nonuniform electric field substantially prolong the kinetics when compared to the uniform field scenario (see Supplementary Movie S4).

**Figure 4:**
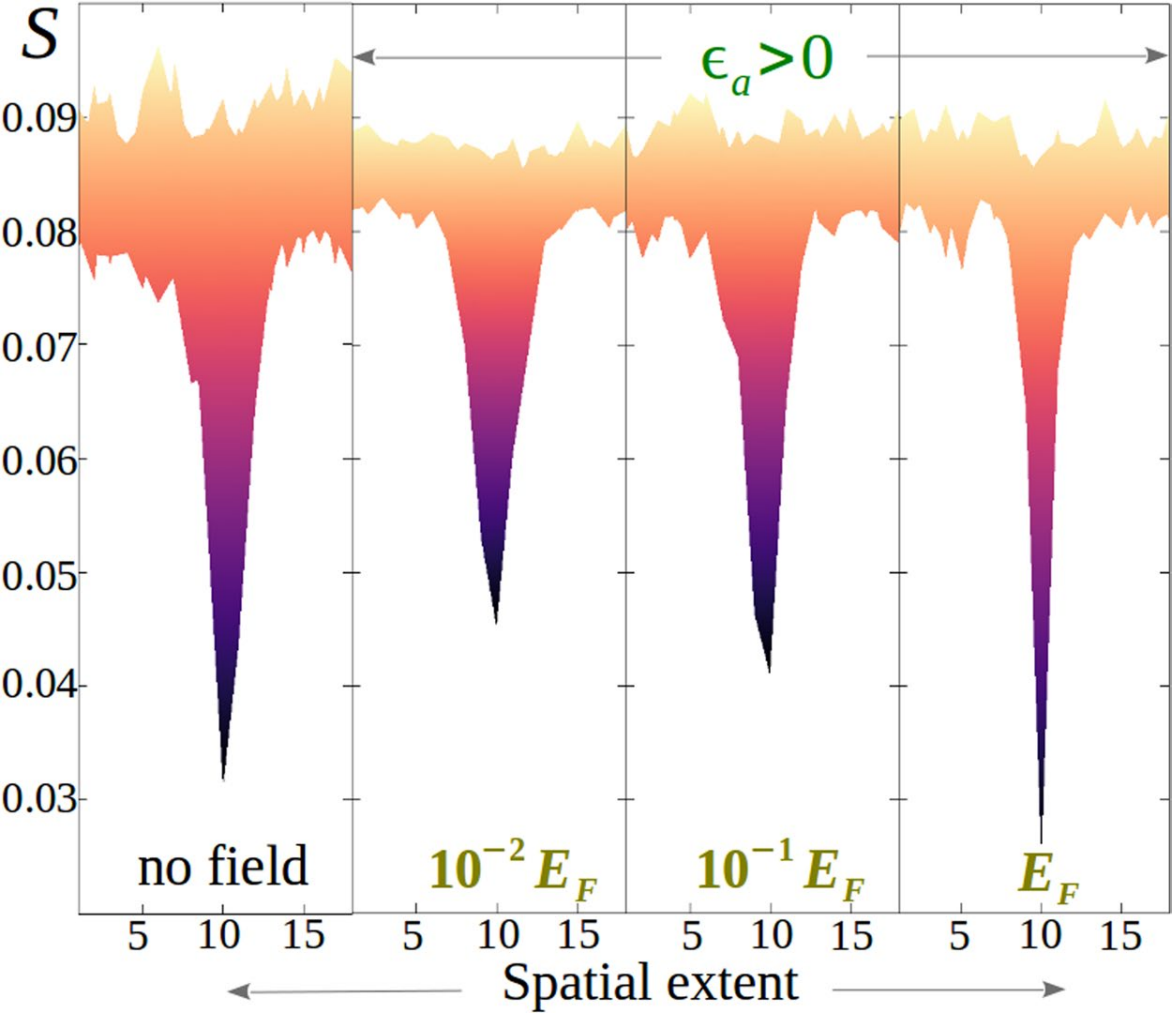
Spatial extent of surfaces of isolated uniaxial planar disclinations obtained from the portion of the *xy*-slice plane of the three-dimensional volume in Fig. 2 (frame b). Colourbars are indicated in Fig. 1. Note the reduced fluctuation amplitude of *S* for |*E*| ≠ 0 compared to no field defect core.

To shed light on the effect of electric forces on the disclination core structure in thermal uniaxial media with *ε*_*a*_ > 0, in Fig. 4 we sketch the spatial variation of the surface of *S* around a planar defect for different field intensity and compare with the equilibrium scenario. The fluctuation amplitude at *S*_*ueq*_ is reduced due to the application of an electric field, resulting in a reduction of the disclination surface density (Fig. 3, frame g). However, we do not find any significant distortion of the core for an increasing field strength which indicates that the field, unlike the director orientation, cannot influence the sufficiently isotropic core other than a complete melting of the disclination at $E\gg {E}_{F}$.

### Thermoelectrokinetic effects in coarsening biaxial NLC

Next, we examine the role played by the isotropic elasticity of the medium and various external forcing (*k*_*B*_*T*, *E*) on the kinetics and microstructure of the string disclination assembly of different homotopy class. We do not find excitingly different outcome when investigating the role of anisotropic elasticity and, thus, here we restrict ourselves in reporting results in *κ* = 0 limit, in par with other investigations^16,32^.

#### Role of thermal fluctuations

**Figure 5:**
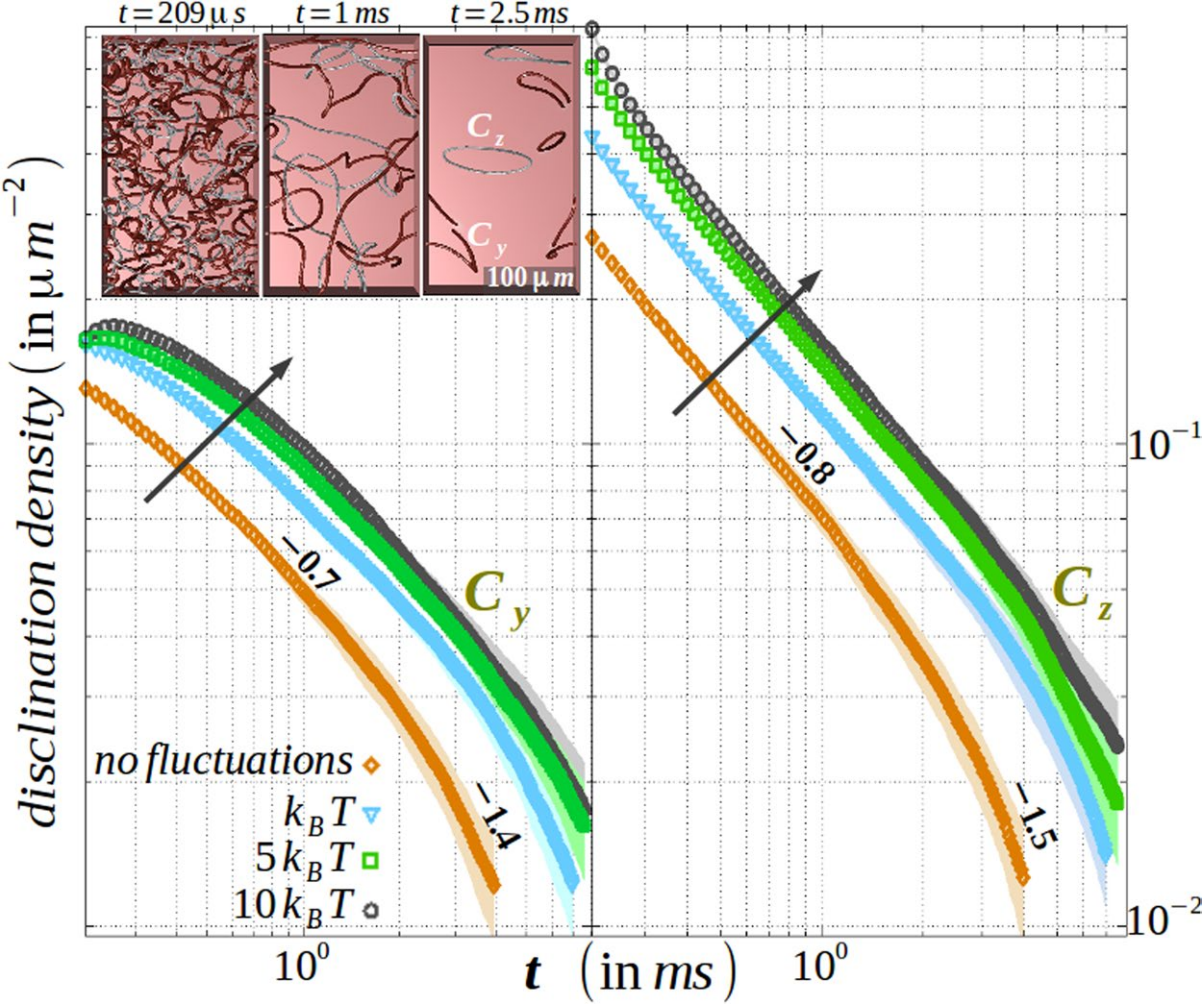
Evolution of disclination density of Cy class (left panel) and Cz class (right panel) with fluctuation amplitude for biaxial nematic media is displayed. The direction of the arrow shows the increment of surface density with fluctuation amplitude. In the inset to the left panel, biaxial order B2 is sketched in which nucleation, intercommutation, and extinction by ring formation of disclinations is portrayed for clarity. Slope and errorbar are indicated within the graphics. Material (computation) parameters are tabulated in Table 1.

We estimate the consequence of thermal fluctuations on the biaxial disclinations of class {*C*_*y*_, *C*_*z*_}. In Fig. 3, we plot the evolution of disclination density for different values of *k*_*B*_*T* and compare with the athermal scenario. To remind, in the inset to the left panel, we portray the early, intermediate and late stage of the disclination kinetics. Similar to the uniaxial disclinations, here we also find that thermal fluctuation increases the disclination density per unit area with a comparable slope. The increase in slope for an increase in *k*_*B*_*T* during early stage of the kinetics for *C*_*y*_ class hints for a delayed emergence of Porod’s regime, that is absent in *C*_*z*_ class. More interestingly, we observe an equivalence of the *C*_*z*_ class of biaxial disclinations with that of the ±1/2-integer disclinations in uniaxial nematics, both (i) in morphology (Fig. 1: frame c and frame g) and (ii) kinetics, as observed in the identical slope of 0.8 in the Porod’s law scaling regime (Fig. 5: right panel and Fig. 2: frame a). Also, the mismatch of the slope between the *C*_*y*_ and *C*_*z*_ class of disclinations (Fig. 5) suggests of minor influence within each other in the course of annihilation.

#### Role of an electric field

**Figure 6:**
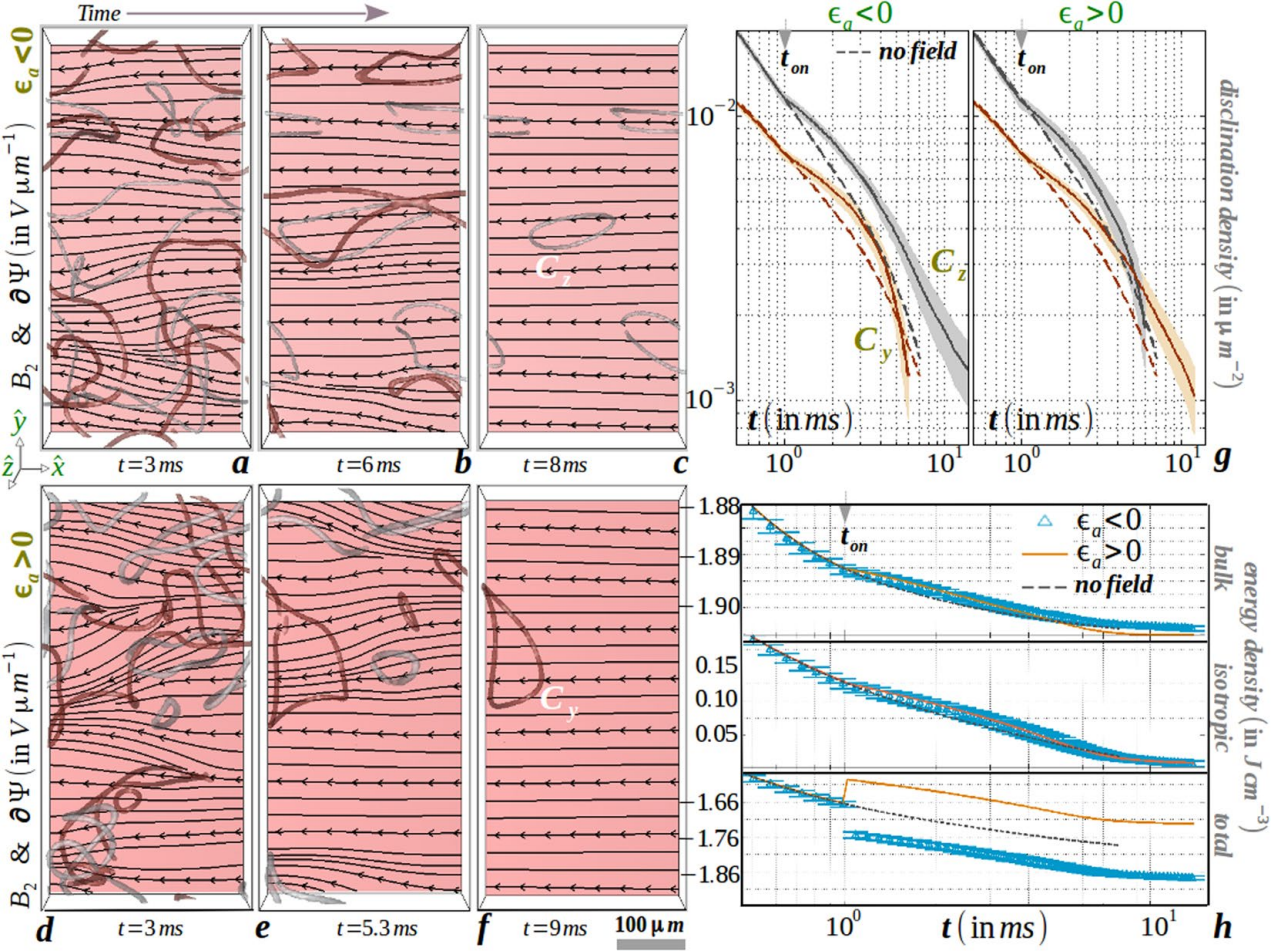
(**a**–**c**) Evolution and selection of disclinations of homotopy class *C*_*z*_ for a negative dielectric anisotropy constant material under the onset of an applied field *E* = 1.5 *V*/*μm*. The presence of *C*_*y*_ disclinations at the early stage severely distorts the electric flux lines within the mesogen which regain uniformity as these disclinations are expelled from the medium (see Supplementary Movies S5, S6). (**d**–**f**) Similar response as previous for a positive dielectric anisotropy constant material, except that *C*_*y*_ disclinations are selected and at an early stage, *C*_*z*_ disclinations widely distort the electric flux lines which gain uniformity after their expulsion. (**g**) Decay of disclination surface density to portray quantitatively the selection of disclination class and (**h**) contributions from volume and surface energy to the total (free) energy of the medium. Equilibrium response is also sketched for comparison. Material (computation) parameters are tabulated in Table 1.

To conclude, we examine the thermal and electrokinetic effects on the coarsening of biaxial NLC when the sample is rapidly cooled from a disordered phase in the presence of a steady voltage pulse. Figure 6 plots the instantaneous snapshots as well as the kinetic evolution of the medium. Although the topological structure and kinetic pathway of disclinations in biaxial NLC have been predicted for long^1,18,33^, experimental advance to stabilize disclinations by avoiding crystallization continues to be the *holy grail* of research on thermotropic biaxial mesogens^34^. Similar to the dilated kinetics of electrically forced uniaxial disclinations (see Fig. 3), we find in Fig. 6 (frame g) that the onset of an electric field increases the lifetime of biaxial disclinations of homotopy class {*C*_*y*_, *C*_*z*_} at the initial stage for both sign of dielectric anisotropy. For either class of disclinations, *ν*(= *πε*_0_*ε*_*a*_*E*^2^/8*kη*) >0 qualitatively explains the slowing down. For both signs of the dielectric anisotropy constant of the material, flux lines are massively distorted in the presence of disclinations (frames a,d). After an interval of ~5 *ms*, a clear asymmetry between the disclination kinetics of different topology becomes evident. As shown in frames b,c,e,f,g, this results in long-lived disclinations of either class with uniform electric field lines. This is attributed to an increment (decrement) of the total free energy with a positive (negative) value of the dielectric anisotropy constant (frame h). Thus, the dielectric energy has a strong influence in selecting disclinations of the desired class as the bulk and elastic energies increase negligibly from the no field scenario. Physically, the acceleration (or retardation) in the loop extinction kinetics at the late stage can be interpreted as effective acceleration (or retardation) in speed of approach between ±1/2-charged topological dipole within a charge-neutral loop of different class due to the electric force. Consistent evidence of class selection is also obtained, however on a much longer timescale, for values of the electric field magnitude much smaller than the Fréedericksz threshold value. However, the decay kinetics of disclinations of a particular homotopy class is accelerated in the presence of an intense electric field due to the absence of backflow in our model to compensate the electric drag. We expect new phenomena in experiments on thermotropic biaxial media under an intense electric field, perhaps similar to the behavior of uniaxial disclinations under an intense electric field^6^.

## Discussions

Electrorheology of line defects, with^35–37^ or without^23^ particulate inclusion, under an intense electric field have established the coupling of orientation tensor with hydrodynamics and uniformity in the electric field, though the effect of nonuniformity in the electric field^19,38–40^ and the effect of thermal fluctuations are less explored. The effect of hydrodynamics is assumed to be negligible under moderate to weak electric field intensity^41^. We have examined the role of thermal fluctuations and nonuniformity of electric field in this limit and have shown that fluctuating electronematics is a robust tool to mimic laboratory experiments^6,7,13^ *in silico* for anisotropic NLC in three dimensions. From the structure of the orientation tensor, we present a simple way to identify and classify the line defects and to compute physical quantities from the geometry of disclinations.

We have shown how the spatial uniformity in electric field is gained in approaching the Fréedericksz limit. Apart from modifying the kinetic pathway of the coarsening of athermal disclination network, the external stimuli in terms of temperature fluctuations and local electric field essentially probe two emergent length scales: (i) interfacial correlation length between isotropic and nematic phase and (ii) radius of curvature of disclination loop. Neglecting any local heating effects due to the variation of temperature, any change in the correlation length is attributed to the disclination core size as well its geometric position within the three-dimensional volume. Although the evolution is temporally dilated compared to the no-field scenario, the external field cannot sufficiently modify the radius of curvature of isotropic disclinations - thus the lines are not stretched along the direction of the electric field, rather they retain their shape even when the director gets aligned along or perpendicular to the field direction depending on the sign of material’s dielectric anisotropy. The inhomogeneity of the nematic orientation is manifest in the inherent nonuniformity of the local electric field - resulting in the highly nonuniform electric flux lines within the sample. The electric field induces a memory to the material that exhibit an elastic response and also induces a kinetic asymmetry within disclinations of the different class. On the other hand, increase in thermal fluctuations tends to increase the disclination surface density.

This complex interaction can be intelligently engineered to yield a fascinating outcome in a more complex scenario, for example, fractal nematic colloids^42^, metadevices^43^ and photonic applications^44^. The electric field induced kinetic asymmetry leading to the class selection of biaxial disclinations can develop into novel materials in topologically similar systems. Other than NLC, the presented work has resemblance with line defects in passive^2,3,9^ and active^10,45^ soft matter including conducting microwires^46^ and self-assembled resonators^47^, and thus has the potential to bring exciting applications in diverse systems.

## Methods

### Fluctuating electronematics: model energy and thermal kinetics

Instantaneous orientational order, that distinguishes between the disordered liquid state and partially ordered nematic state, is characterized by a symmetric traceless second rank tensor^1^

2 $${\bf{Q}}=\frac{1}{2}[3S\,\,\overline{{\bf{n}}{\bf{n}}}\quad +{B}_{2}({\bf{l}}{\bf{l}}-{\bf{m}}{\bf{m}})];\,-\frac{1}{3}\le S\le \frac{2}{3},\,{B}_{2}\, < \,3S,$$

where {n,l,m} denote the alignment direction of the long (director), intermediate (codirector) and short axis (secondary director) with the degree of uniaxiality $S\propto {\langle {Y}_{2}^{0}\rangle }_{t}$ and biaxiality ${B}_{2}\propto {\langle Re[{Y}_{2}^{\pm 2}]\rangle }_{t}$. ${Y}_{l}^{m}$ are the spherical harmonics of degree *l* with order *m*, 〈⋅〉_*t*_ denotes the ensemble average at that instant *t* and symmetric traceless is symbolized with$^{\prime} \bar{\cdot }^{\prime}$. While *B*_2_ cease to null as *S* attains its maximum, other moments *R*_2_ generated from the planar projection of {n, l} is also exercised to define biaxiality^33^. *B*_2_ is numerically less expensive in considering one less degree of freedom, nevertheless, *R*_2_ plays an important role in understanding the field-induced switching kinetics^48^.

The ground state free energy including the excitations due to the spatial distortions and dielectric coupling are represented in the phenomenological Ginzburg-Landau-de Gennes (GLdG) free energy functional

3 $${ {\mathcal F} }_{total}={\int }_{{\mathbb{V}}}{d}^{3}x({ {\mathcal F} }_{bulk}\,[{\bf{Q}}]+{ {\mathcal F} }_{elastic}\,[{\boldsymbol{\partial }}Q]+{ {\mathcal F} }_{dielec}\,[{\bf{Q}}]),$$

where ${\mathbb{V}}$ is the material volume. Bulk energy is superposition of absolute rotationally invariant functions of tensorial order ${{\mathscr{F}}}_{bulk}\,[{\bf{Q}}]=A{\rm{T}}{\rm{r}}{{\bf{Q}}}^{2}/2+B{\rm{T}}{\rm{r}}{{\bf{Q}}}^{3}/3+C{({\rm{T}}{\rm{r}}{{\bf{Q}}}^{2})}^{2}/4+E{\rm{^{\prime} }}{({\rm{T}}{\rm{r}}{{\bf{Q}}}^{3})}^{2}$, where parameters {*A*,*B*} control the system temperature and size disparity, *C* > 0 preserves the boundedness and *E*′ ≠ 0 brings the notion of biaxiality^1^. Higher order expansions are not required while^32^ (Tr**Q**^2^)^3^/6 ≥ (Tr**Q**^3^)^2^. Order in equilibrium is obtained from ${\partial }_{S}{ {\mathcal F} }_{bulk}\,(S)={\partial }_{{B}_{2}}{ {\mathcal F} }_{bulk}\,({B}_{2})=0$, which for a uniaxial media is *S*_*ueq*_ =−*B*/6*C* + (*B*^2^/36*C*^2^ −2*A*/3*C*)^1/2^; (*B*_2_)_*ueq*_ = 0, while for a biaxial media, ${({B}_{2})}_{beq}={[-2A+{S}_{beq}\mathrm{(2}B-3C{S}_{beq}+9E^{\prime} {S}_{beq}^{3})]}^{\mathrm{1/2}}/[C+9E^{\prime} {S}_{beq}^{2}{]}^{\mathrm{1/2}}$ and clumsy algebraic expression for *S*_*beq*_ is omitted for brevity.

Inhomogeneities due to the excitations above the ground state is concealed within the symmetry allowed lowest order terms in ${{\mathscr{F}}}_{elastic}[{\boldsymbol{\partial }}{\bf{Q}}]=[{L}_{1}{({\boldsymbol{\partial }}{\bf{Q}}{\boldsymbol{)}}}^{2}+{L}_{2}{({\boldsymbol{\partial }}\cdot {\bf{Q}})}^{2}+{L}_{3}{\bf{Q}}\cdot {({\boldsymbol{\partial }}{\bf{Q}}{\boldsymbol{)}}}^{2}]/2$, where ratio *κ* = *L*_2_/*L*_1_ and Θ = *L*_3_/*L*_1_ of the elastic constants can be mapped to the Frank constants splay, bend and twist^49^. For *MBBA*, (*κ*,Θ) ≈ 1 while for 5*CB*, *κ* ≈ 40,Θ ≈ 1 which designate nearly equal splay and bend constants for both materials (Θ ≠ 0) but the twist constant is an order smaller than splay or bend. This results into nucleation of integer topological charged nematic droplets in the metastable isotropic medium of 5CB, while topologically uncharged nematic droplets nucleate in the metastable medium of MBBA^20,50^.

The optical dielectric permittivity tensor is related to the orientation tensor when separated into symmetric and antisymmetric part ***ε*** = *ε*_*s*_***δ*** + *ε*_*a*_**Q**, where ***δ*** is the Kronecker delta, *ε*_*s*_ = Tr***ε***/3 and ${\varepsilon }_{a}=\mathrm{2(}{\varepsilon }_{\parallel }-{\varepsilon }_{\perp }\mathrm{)/3}$ with ${\varepsilon }_{\parallel }({\varepsilon }_{\perp })$ being the permittivity along (orthogonal to) the director. The material parameters are *ε*_*s*_ = 0.74*ε*_*a*_, where for 5*CB ε*_*a*_ = 5.8 at *T* = 33.65 °C and *ε*_*a*_ = −0.7 for *MBBA* at *T* = 25 °C^51,52^. Application of a spatially varying electric field **E** **=−∂**Ψ leads to an electric displacement **D** **=** **ε** ⋅ **E** and therefore to a dielectric (free) energy term ${ {\mathcal F} }_{dielec}[{\bf{Q}}]=-{\varepsilon }_{0}{\bf{D}}\cdot {\boldsymbol{\partial }}{\rm{\Psi }}\mathrm{/8}{\boldsymbol{\pi }}$ with *ε*_0_ being the vacuum permittivity and Ψ the electric potential. We characterize the intensity of the electric field with respect to the order^19,53^ and thermal energy by the nondimensional ratios *ε*_1_ = (*ε*_0_*ε*_*a*_*E*^2^/8*πAS*_*ueq*_)^1/2^ and *ε*_2_ = (*ε*_0_*ε*_*a*_*E*^2^/8*πk*_*B*_*T*)^1/2^. We estimate the Fréedericksz threshold to orient a director in a uniformly oriented nematic state along (orthogonal to) an electric field in a twist geometry, calculated by minimizing the free energy for director distortion and field coupling, to be^54^

4 $${E}_{F}=\frac{\pi }{{L}_{x}}{[\frac{9{S}^{2}{L}_{1}\mathrm{\{1}+\frac{2}{3}(\kappa +{\rm{\Theta }})\}}{2{\varepsilon }_{0}{\varepsilon }_{a}}]}^{\mathrm{1/2}};\,{{\rm{\Psi }}}_{F}={L}_{x}{E}_{F},$$

where *L*_*x*_ is the spacing between the electrodes.

In the present study, confinement effects *e*.*g*. centrosymmetry breaking geometric restriction at the boundary by coverslips, patterned or chemically active walls, curvature induced polarization and the presence of free ions are not considered. For bent-core molecules, curvature induced polarization can be incorporated by adding ***P***_*f*_ = *c*_1_**∂** ⋅ **Q** + *c*_2_∂ ⋅ (**Q**·**Q**) + *c*_3_**∂**(Tr**Q**^2^) + *c*_4_[**Q** ⋅ (∂ **Q**) − (**Q** ⋅ **∂**) ⋅ **Q**] to the electric displacement **D**, where *c*_1,…,4_ are coefficients^55^. Free ions can also be neglected by retaining $n\ll {\varepsilon }_{0}{{\rm{\Psi }}}_{F}/e{L}_{x}^{2}$, where *n* is the free ion density and *e* the electric charge^19^.

When the medium is sufficiently dry so that the long ranged hydrodynamic interaction produced by the motile disclinations do not interfere the kinetics and fluid inertia plays no role - thus restricting to an overdamped relaxational kinetics without convection of momentum, the Langevin equation displaying the time evolution of the electric potential together with the orientation tensor can be written as

5 $${\partial }_{t}{\rm{\Psi }}={\boldsymbol{\partial }}\cdot {\bf{D}};\,{\partial }_{t}{\bf{Q}}=-{\boldsymbol{\Gamma }}:\,\frac{\delta { {\mathcal F} }_{total}}{\delta {\bf{Q}}}+{\boldsymbol{\xi }},$$

where **Γ **= Γ[*δ*_*ik*_*δ*_*jl*_ + *δ*_*il*_*δ*_*jk*_ − 2*δ*_*ij*_*δ*_*kl*_/3] is a 4^*th*^ rank tensor that maintains the symmetric-traceless property on the right-hand side of second equation (5). The rotational diffusion constant Γ is approximated to be independent of **Q** and the stochastic term *ξ* satisfies the property of the orientation tensor 〈*ξ*(x, *t*)〉 = 0, 〈*ξ*(x, *t*)*ξ*(x′, *t*′)〉 = 2*k*_*B*_*T***Γ***δ*(*x* − *x*′)*δ*(*t* − *t*′) and is constructed as a summation of Wiener process to keep discrete fluctuation dissipation (FDT) spectrum intact over all Fourier modes and thus to sample Gibbs distribution in thermal equilibrium^26^. We stress at this point that the functional derivative of ${ {\mathcal F} }_{dielec}[{\bf{Q}}]$ with respect to Ψ is the divergence of the electric displacement **D**^38^. This legitimate the imposition of Maxwell’s equation along with the **Q**-tensor equation. Unlike ref.^40^, we neglect cross-coupling between terms proportional to $\delta {{\mathscr{F}}}_{total}/\delta \psi$ to the right-hand side of ∂_*t*_*Q* equation and $\delta {{\mathscr{F}}}_{total}/{\boldsymbol{\delta }}{\bf{Q}}$ to the right-hand side of ∂_*t*_*ψ* equation for simplicity.

By substituting equation (3) in equation (5), the coupled Maxwell-GLdG equation in expanded form reads

6 $$\begin{array}{rcl}{\partial }_{t}{\rm{\Psi }} & = & {\varepsilon }_{0}({\varepsilon }_{s}\,{\partial }^{2}{\rm{\Psi }}+{\varepsilon }_{a}{\partial }_{i}{Q}_{ij}{\partial }_{j}{\rm{\Psi }}),\\ {\partial }_{t}{Q}_{ij} & = & -{\rm{\Gamma }}[(A+C{{\rm{Tr}}{\bf{Q}}}^{2}){Q}_{ij}+(B+6E^{\prime} {{\rm{Tr}}{\bf{Q}}}^{3})\,\bar{{Q}_{ij}^{2}}\\  &  & -{L}_{1}\{{\partial }^{2}{Q}_{ij}+{\rm{\Theta }}\,\,\overline{({Q}_{mn}{\partial }_{m}{\partial }_{n}{Q}_{ij}-\frac{1}{2}{\partial }_{i}{Q}_{qr}{\partial }_{j}{Q}_{qr})}\\  &  & +\kappa \,\overline{{{\rm{\partial }}}_{i}{{\rm{\partial }}}_{l}{Q}_{jl}}\,\}\,-\frac{1}{8\pi }{\varepsilon }_{0}{\varepsilon }_{a}\,\overline{{\partial }_{i}{\rm{\Psi }}{\partial }_{j}{\rm{\Psi }}}]+{\xi }_{ij}\mathrm{.}\end{array}$$

A significant departure from uniformity in electric flux lines is expected when ${\mathscr{O}}({\rm{\Psi }}/{{\rm{\Psi }}}_{F}) \sim {\mathscr{O}}({\bf{Q}})$. Thus, near and below the supercooling temperature *T* < T^*^, the orientational order is small and is affected by a moderate to small magnitude of the electric force. Conversely, under an intense electric field $E\gg {E}_{F}$, ${\mathscr{O}}({\rm{\Psi }}/{{\rm{\Psi }}}_{F})\gg {\mathscr{O}}({\bf{Q}})$ and the electric field can remain spatially uniform by decoupling from the *Q*-tensor equation (6) to yield,

7 $$\begin{array}{ccc}{{\rm{\partial }}}_{t}{Q}_{ij} & = & -{\rm{\Gamma }}[(A+C{\rm{T}}{\rm{r}}{{\bf{Q}}}^{2}){Q}_{ij}+(B+6E{\rm{^{\prime} }}{\rm{T}}{\rm{r}}{{\bf{Q}}}^{3})\bar{{Q}_{ij}^{2}}\\  &  & -{L}_{1}\{{{\rm{\partial }}}^{2}{Q}_{ij}+{\rm{\Theta }}\,\overline{({Q}_{mn}{{\rm{\partial }}}_{m}{{\rm{\partial }}}_{n}{Q}_{ij}-\frac{1}{2}{{\rm{\partial }}}_{i}{Q}_{qr}{{\rm{\partial }}}_{j}{Q}_{qr})}\\  &  & +\kappa \overline{{{\rm{\partial }}}_{i}{{\rm{\partial }}}_{l}{Q}_{jl}}\}-\frac{1}{8\pi }{\varepsilon }_{0}{\varepsilon }_{a}\,\overline{{E}_{i}{E}_{j}}]+{\xi }_{ij}.\end{array}$$

An extensive numerical route of investigation is presented next.

The deterministic part of the equation (7) has been widely exercised in two-dimensional monolayered thin films in one elastic constant approximation (*κ* = Θ = 0) to examine electrokinetic effects of point defects in switching experiments^27,56^. However, an impeccable role of backflow and elastic anisotropy is found to decipher asymmetric speed of ±1/2 integer defects under intense electric field^23,30^, that also leads to ceasing motility of disclinations due to the backflow^6^. A quantitative measure in three-dimensional media under intense electric field, albeit experimentally posed for single disclination in deep uniaxial state^6^, is yet to be sought by including Beris-Edwards model^41^ for fluid flow to the presented fluctuating electronematics model. Here instead, we focus on moderate to the small magnitude of electric forces including thermal fluctuations at temperature close and below T^*^ where advective effects can be neglected for simplicity.

### Stochastic method of lines for fluctuating electronematics

We consider a thick rectangular slab of insulating thermotropic NLC material in thermal equilibrium, with an 80 *μm* × 160 *μm* base and 80 *μm* height. At equilibrium, periodic boundaries in three Cartesian directions are retained, that can be realized as a free standing anisotropic thick film from a groove. The electric potential at *x* = 0 is fixed at zero and at *x* = *L*_*x*_ is held according to the desired field magnitude with Dirichlet boundary condition and periodicity is maintained in the *yz*-directions. This is mimic by suspending the thick slab within two planar laser beams kept at a different potential or placing within an electrode without the notion of an *easy axis*.

An elegant way to numerically integrate equations (6,7) while retaining the symmetric-traceless property of {*Q*, *ξ*} tensor is by projection on a basis of five 3 × 3 matrices as^26,57^ Q = ∑_*l*_*a*_*l*_T_*l*_;*ξ* = ∑_*l*_*ζ*_*l*_T_*l*_ (*l* = 1, …, 5), so that the kinetics is projected into the basis coefficients *a*_*l*_. The fluctuating force in the projected basis has the property, 〈*ζ*_*l*_(*x*, *t*)〉 = 0,〈*ζ*_*l*_(x, *t*)*ζ*_*m*_(x′, *t*′)〉 = 2*k*_*B*_*T*Γ*δ*_*lm*_*δ*(x − x′)*δ*(*t* − *t*′). After projection, equation (6) translates into

8 $$\begin{array}{ccc}{{\rm{\partial }}}_{t}{\rm{\Psi }} & = & {\varepsilon }_{0}({\varepsilon }_{s}\,{{\rm{\partial }}}^{2}{\rm{\Psi }}+{\varepsilon }_{a}{T}_{ij}^{l}{{\rm{\partial }}}_{i}{a}_{l}{{\rm{\partial }}}_{j}{\rm{\Psi }}),\\ {{\rm{\partial }}}_{t}{a}_{l} & = & -{\rm{\Gamma }}[(A+C{\rm{T}}{\rm{r}}{{\bf{Q}}}^{2}){a}_{l}+(B+6E{\rm{^{\prime} }}{\rm{T}}{\rm{r}}{{\bf{Q}}}^{3}){T}_{ij}^{l}\bar{{Q}_{ij}^{2}}\,\\  &  & -{L}_{1}\{{{\rm{\partial }}}^{2}{a}_{l}+{\rm{\Theta }}({Q}_{mn}{{\rm{\partial }}}_{m}{{\rm{\partial }}}_{n}{a}_{l}-{T}_{ij}^{l}{{\rm{\partial }}}_{i}{a}_{p}{{\rm{\partial }}}_{j}{a}_{p})\\  &  & +\kappa \,\overline{{T}_{ij}^{l}{T}_{jk}^{p}{{\rm{\partial }}}_{i}{{\rm{\partial }}}_{k}{a}_{p}}\,\}-\frac{1}{8\pi }{\varepsilon }_{0}{\varepsilon }_{a}\overline{{T}_{ij}^{l}{{\rm{\partial }}}_{i}{\rm{\Psi }}{{\rm{\partial }}}_{j}{\rm{\Psi }}}\,]+{\zeta }_{l},\end{array}$$

while equation (7) takes the form,

9 $$\begin{array}{ccc}{{\rm{\partial }}}_{t}{a}_{l} & = & -{\rm{\Gamma }}[(A+C{\rm{T}}{\rm{r}}{{\bf{Q}}}^{2}){a}_{l}+(B+6E{\rm{^{\prime} }}{\rm{T}}{\rm{r}}{{\bf{Q}}}^{3}){T}_{ij}^{l}\,\bar{{Q}_{ij}^{2}}\\  &  & -{L}_{1}\{{{\rm{\partial }}}^{2}{a}_{l}+{\rm{\Theta }}({Q}_{mn}{{\rm{\partial }}}_{m}{{\rm{\partial }}}_{n}{a}_{l}-{T}_{ij}^{l}{{\rm{\partial }}}_{i}{a}_{p}{{\rm{\partial }}}_{j}{a}_{p})\\  &  & +\kappa \,\,\overline{{T}_{ij}^{l}{T}_{jk}^{p}{{\rm{\partial }}}_{i}\,{{\rm{\partial }}}_{k}{a}_{p}}\,\}-\frac{1}{8\pi }{\varepsilon }_{0}{\varepsilon }_{a}{T}_{ij}^{l}\,\overline{{E}_{i}{E}_{j}}\,]+{\zeta }_{l}.\end{array}$$

To numerically integrate the above equations (8 or 9), we adopt a central finite differencing to spatially discretize the Laplacian and mixed derivates. An explicit stochastic method of lines (SMOL) integrator is exercised for seamless temporal integration^58^. SMOL is a stochastic generalization of the deterministic method of lines approach^59^ that relies on discretizing the spatial derivates without a temporal discretization, thus yielding to a set of ordinary differential equations in time, that can be easily integrated using the standard numerical libraries^60^. Though spatial accuracy can be increased by using spectral collocation method^61^, obtained solution is usually limited by the temporal accuracy of the integrator. SMOL is numerically stable without computational hindrance with convergence, is less computationally overloaded, spatiotemporally second order accurate, satisfies discrete FDT and can faithfully reproduce lab-based experiments *in silico*^7,13,20,29,30^.

In coarsening kinetics, a kinetic length scale is extracted from the scattered light intensity inscribed within the structure function *S*(q,*t*) as *ξ* = [∫*d*^3^q*S*(q,*t*)*q*^2^/∫*d*^3^q*S*(q,*t*)] ^−1/2^, where *S*(q, *t*) = *Q*_*ij*_(**q**, *t*)*Q*_*ij*_(−*q*, *t*)/∫*d*^3^*qQ*_*ij*_(**q**, *t*)*Q*_*ij*_(−**q**, *t*) with the orientation tensor defined as ${\bf{Q}}({\bf{q}},t)={\int }_{{\mathbb{V}}}{d}^{3}x{\bf{Q}}({\bf{x}},t){e}^{-i{\bf{q}}\cdot {\bf{x}}}/{\mathbb{V}}$^62^. At equilibrium, the Ornstein-Zernicke form of the spatial correlation 〈**Q**(0)**Q**(**x**)〉 ∝ **Γ***e*^−|x|/*ζ*^/|x| defines the coherence length *ζ* = [{1 + 2(*κ* + Θ)/3}*L*_1_/*A*]^1/2^. By definition, *ζ* determines the core size of a disclination which is distinct from the length scale *ξ* that denotes the mutual separation between two strings and also the separation between ±1/2 integer dipoles within a shrinking disclination loop (or the radius of curvature). Thus, the length scale obtained from the disclination surface density $\rho  \sim {\xi }^{-2}$ shown in frame 3 g is identical with the length scale obtained from correlation functions^16^. In our numerics, time, length, and energy scales are resolved by non-dimensionalizing equations (8,9)^20^ and strictly retaining (i) Courant-Friedrichs-Lewy condition for timestep to avoid stiffness^63^, (ii) ${\rm{\Delta }}x\ll \zeta$ to resolve the grid spacing and distortion length embedded in the disclination core, (iii) **∂***S* < *S*/*ζ* for the validity of the GLdG method and, (iv) ${k}_{B}T\ll$ barrier height between isotropic-nematic state at clearing point to avoid any spurious oscillations between the two phases, thus, ${k}_{B}T={ {\mathcal F} }^{\ast }\mathrm{/10}$, where ${ {\mathcal F} }^{\ast }=9C{S}_{ueq}^{4}\mathrm{/16}$ is the non-dimensionalized distortion free energy^16^. With the chosen values of {*ε*_0_, *ε*_*s*_, *ε*_*a*_}, electric field relaxation is rapid compared to the orientational kinetics, so as a steady electric field is obtained for each step of **Q**-evolution. Grid size independence, numerical accuracy, and validity of physical tests were confirmed for each presented graphics and for both equations (8,9).

We finally discuss difference of our method with existing methodology in connection with the electrorheology of disclinations. Within Leslie-Ericksen (LE) theory in one dimension using free energy minimization technique, both the effect of rheology^64^ and nonuniform electric field^40^ has been exercised. Traditionally, LE theory is suitable in deep within the nematic phase where it is assumed that {*S*, *B*_2_, **l**} are constant in the orientation tensor and elastic distortion is concealed in **n** only. Earlier attempts to include hydrodynamics^65^ to LE theory were through the finite element method (FEM)^66^. Also, the finite volume method (FVM) applied to Smoluchowski equation for the orientational probability density function is yet at a preliminary stage^67^. On the other hand, after the advent of large scale computation, numerical solution of the athermal orientation tensor equation retaining all independent degrees of freedom is exercised through cell-dynamic scheme (CDS)^18^, finite difference methods (FDM)^35,68^ as well as the method of lines (MOL)^59^ approach to the GLdG theory. Attempts to include hydrodynamics and thermal fluctuations to the FDM via fluid particle dynamics (FPD)^35^ were replaced with the Lattice-Boltzmann method (LBM)^37,69^, while inclusion of thermal fluctuations retaining second-order numerical accuracy is extended as a stochastic generalization of the method of lines (SMOL)^26^. In this manuscript, SMOL complements the inclusion of Maxwell’s equation to the existing formulation and motivates the association of hydrodynamics^24^ for intense electric field studies and Fourier’s law of latent heat conduction^70^ for confined NLC systems.

### Identification and topological classification of disclinations

**Figure 7:**
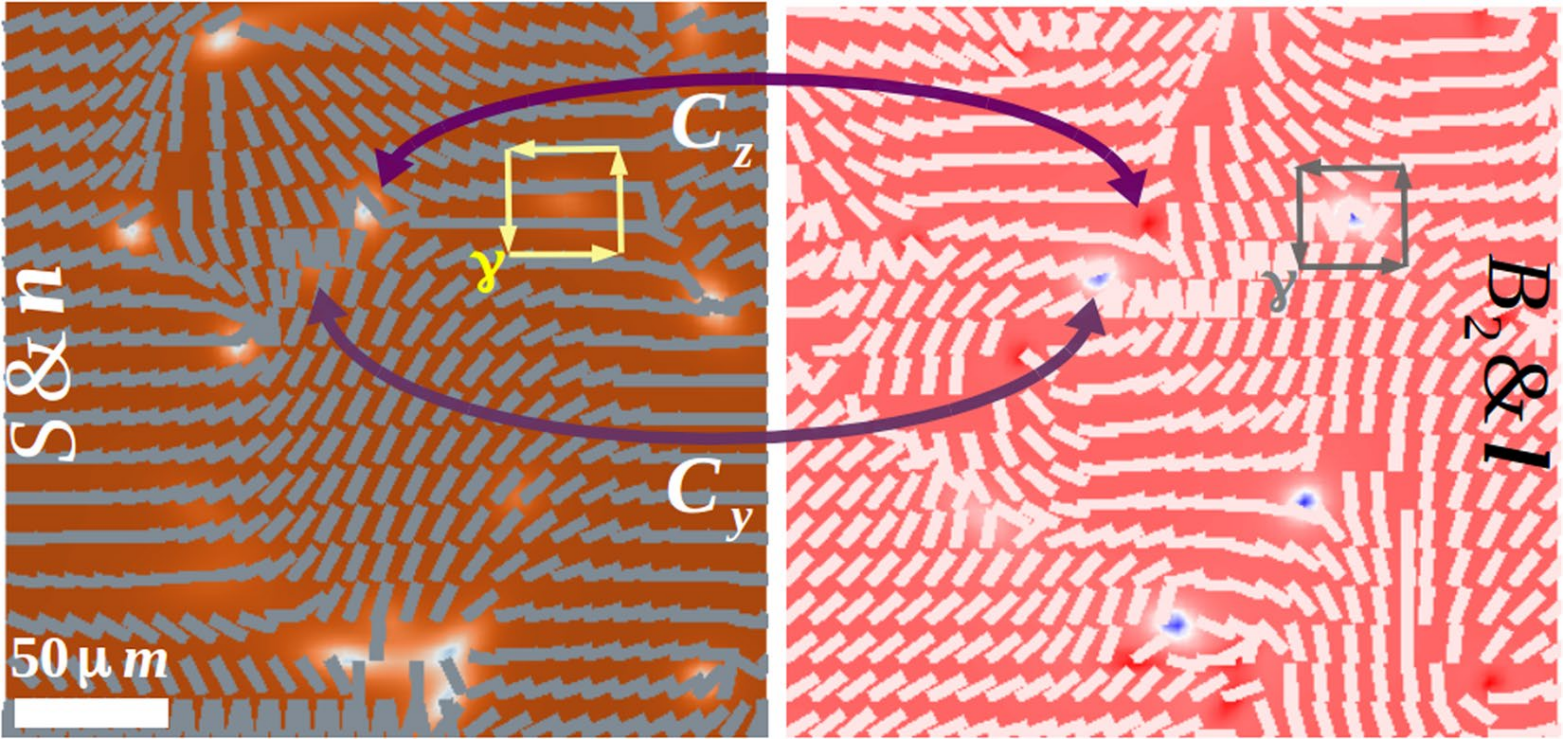
A portion of the *xy*-slice plane of an 80^2^ × 160 *μm*^3^ thermotropic biaxial NLC (portrayed in frame 1 e,g) displaying ±1/2 integer defects corresponding to two different class of disclinations. Note that singular points of homotopy class *C*_*y*_ are markedly different than *C*_*z*_ by **∂*n*** = 0, otherwise for both classes, (∂*S*,∂*B*_2_,∂*l*) ≠ 0. Material (computation) parameters are tabulated in Table 1.

Disclinations of different homotopy class are obtained after extracting {*S*,*B*_2_,n,l} from the basis coefficients *a*_*i*_ on each space point by a similarity transformation. The scalar values are colour rendered according to the indicated bars in Fig. 3. Disclinations are identified by sketching the isosurfaces with specific isovalue of the scalar fields. At a late stage of the kinetics when few isolated disclinations are existent, a plane from the three-dimensional volume is sliced in which the lateral section of disclinations appear as points. Once the physical location of disclinations are identified, the homotopy class, charge and sign of defect is calculated from the spatial distribution of {n,l}. Similar to the Volterra construction in crystal dislocation, a Burgers circuit *γ* displayed in Fig. 7 is constructed using neighboring lattice points that encircle the defect^18^. The angular shift of {n,l} is measured while traversing one complete loop and the charge and sign of defects are estimated using the hodograph method^1^. If *u* = (*sinθ cosϕ*, *sinθ sinϕ*, *cosθ*) is directionally equivalent with −**u** = (*sin****θ***′*cos****ϕ***′, *sin****θ***′*sin****ϕ***′, *cos****θ***′) where (*θ*, *ϕ*) are polar and azimuthal angle in an arbitrary frame, then following transformation retains the centrosymmetry,

10 $$\theta ^{\prime} \to \pi -\theta ;\,\varphi ^{\prime} \to \pi +\varphi \mathrm{.}$$

For ±1/2 integer disclinations in a slice plane of uniaxial NLC shown in frame 1c, while traversing *γ*, n rotates by ± *π*. In biaxial NLC, homotopy classes are identified using the following recipe:^18^ (i) for *C*_*x*_ class of disclinations, **n** rotates by ±*π* but **l** does not rotate, (ii) for *C*_*y*_ class of disclinations, **n** does not rotate but **l** rotates by ± *π* and, (iii) for *C*_*z*_ class of disclinations, both {n, l} rotates by ±*π*. We do not find any *C*_*x*_ class of disclinations, which is consistent with the analytic prediction on two-dimensional nonabelian vortices^17^ and numerical computations^16,18^. This algorithm not only supersedes the traditional defect classifying approaches using vector field, tensor glyph or hyperstreamline seeding through Mueller and Westin matrices^71,72^ but can uniquely determine disclinations from the structure of orientation tensor rather than rely on the vectorial information.

## Electronic supplementary material

Supplementary Information

Supplementary Movie 1

Supplementary Movie 2

Supplementary Movie 3

Supplementary Movie 4

Supplementary Movie 5

Supplementary Movie 6
