# Supplementary material for: Controlling motile disclinations in a thick nematogenic material with an electric field
Source: Sci Rep. 2018 Feb 6;8:2517. doi: 10.1038/s41598-018-19891-0 (PMC5802863; doi:10.1038/s41598-018-19891-0)
Supplement: Supplementary file 1 — Supplementary Information [file 41598_2018_19891_MOESM1_ESM.pdf]

Supplementary Information : Controlling motile disclinations in a thick nematogenic material with an electric field

Amit Kumar Bhattacharjee[1,2,*]

[1]Asutosh College, University of Calcutta, Kolkata 700026, India
[2]Centre for Condensed Matter Theory, Department of Physics, Indian Institute of Science, Bangalore 560064, India
[*]amitb@physics.iisc.ernet.in

Procedure to sample the field variables

As an initial condition, we prepare the isotropic state by drawing S and T randomly from a normal distribution $\mathscr{N}(0, 2k_BT)$ using Box–Muller transform to the standard library routines[1]. We sample $cos\theta$ from a uniform distribution between -1 and 1, ϕ between 0 and 2π to generate director $\mathbf{n}$ and codirector $\mathbf{l}$. The secondary director $\mathbf{m}$ is constructed through the Gram-Schmidt orthogonalization procedure[2]. The thermal fluctuations are incorporated in each numerical step using the basis transformation $\boldsymbol{\xi} \to \zeta$.[3]. After making the transformation $\mathbf{Q} \to a$, we evolve equation(8), presented in the Methods section in the main article, for different initial configurations and switch on and off the electric potential difference at t_{on} and t_{off} to sample the nonequilibrium state space, as the system transforms from an isotropic phase to a nematic phase. Using an inverse transformation $a \to \mathbf{Q}$ and space derivate of the electric potential Ψ, we reconstruct the orientation tensor and electric flux lines at every space point.

Comparison between uniform and nonuniform electric field scenario

In Supplementary Figure S1, we compare the decay of surface disclination density with time for thermal uniaxial media for both sign of dielectric constant for various cases: (i) equilibrium disclination kinetics (electric field $\mathbf{E} = 0$), disclination network under (ii) uniform and (iii) nonuniform $\mathbf{E}$ for equal ($\kappa = \Theta = 0$) and unequal ($\kappa \neq \Theta \neq 0$) elastic constant approximation. Field switch on (t_{on}) and switch off (t_{off}) with an instantaneous snapshot of the uniaxial degree along with the electric flux lines are also embedded in the graphics. Similar to figure 2**c** in the main article, we find that far from the critical line, the response of the disclinations to the electric field for degenerate and non-degenerate elastic constants are similar to statistical averaging. However, in the presence of nonuniform electric field for both degenerate and non-degenerate elastic constant scenario, the disclinations are long-lived.

References

1. Abhyankar, S., Brown, J., Constantinescu, E., Ghosh, D. & Smith, B. F. PETSc/TS: A modern scalable DAE/ODE solver library. Preprint ANL/MCS-P5061-0114, Argonne National Laboratory (2014).

2. Arfken, G. B., Weber, H. J. & Harris, F. E. *Mathematical Methods for Physicists* (Academic Press, Boston, 2013).

3. Bhattacharjee, A. K., Menon, G. I. & Adhikari, R. Fluctuating dynamics of nematic liquid crystals using the stochastic method of lines. *J. Chem. Phys.* **133**, 044112 (2010).

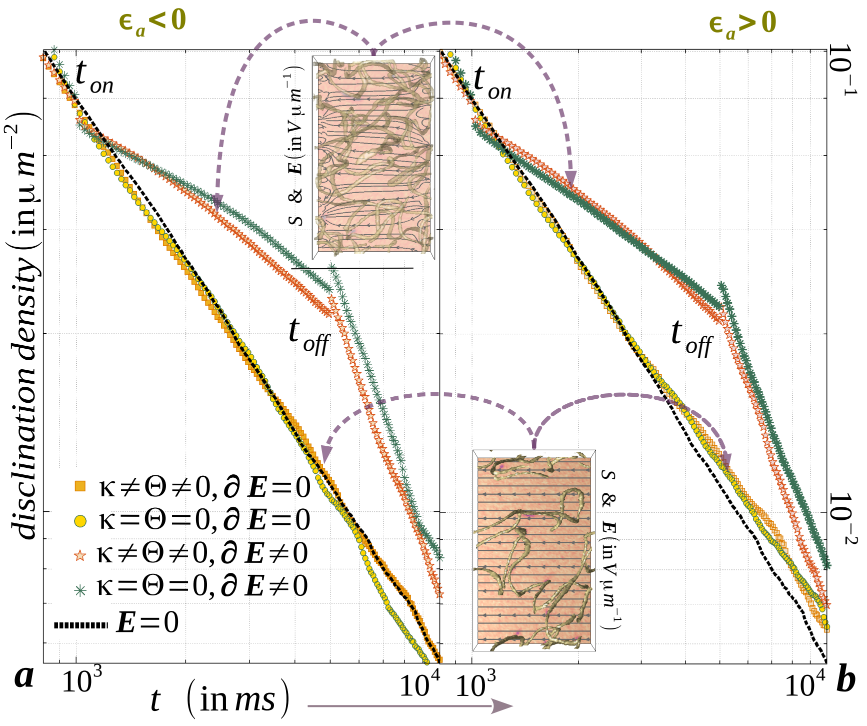

Supplementary Figure S1. Comparison of the kinetics of disclination density per unit area for uniform [solution of equation (7)] and nonuniform [solution of equation (6)] electric field, together with the degenerate (one) elastic constant approximation ($\kappa = \Theta = 0$) and non-degenerate variant ($\kappa \neq \Theta \neq 0$) in a coarsening thermal uniaxial NLC media. Panel **a** (Panel **b**) display the same for negative (positive) dielectric constant material. Though the kinetics for degenerate and non-degenerate elastic constant scenario are very similar, an increased life-span of π-solitons with non-Markovian response during field cessation is observed when nonuniformity in the electric field is considered.

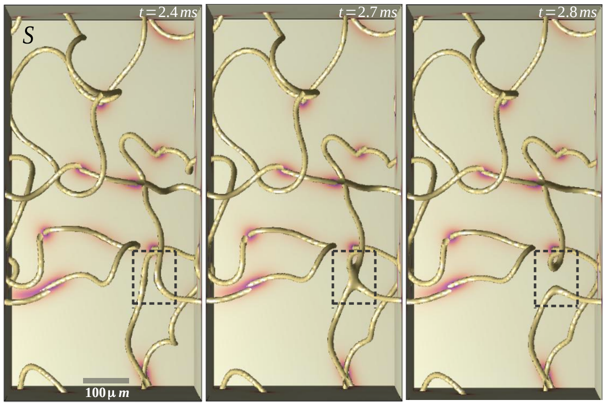

Supplementary Movie S1. Nucleation, intercommutation (marked with dashed box) and extinction of disclinations in a coarsening athermal uniaxial NLC. The animation sequentially portrays of the evolution of uniaxial order S & biaxial order B_2 for an uniaxial NLC subjected to a temperature quench from a disordered isotropic phase to an ordered uniaxial nematic phase. Colourbars and isovalues corresponding to the isosurfaces of disclinations are indicated in figure 1 in the main article. As S grows towards the saturation value S_{ueq}, isotropic domain coarsening leads to the nucleation of charge neutral disclinations with $\pm 1/2$ integer topological dipoles at two end segments at around $1.2ms$. These strings intercommute by exchanging segments with each other to form closed contractile loops that are extinct from the medium to minimize the total (free) energy. The right panel displays of the reduced B_2 in the media with $(B_2)_{max}$ around the disclination core.

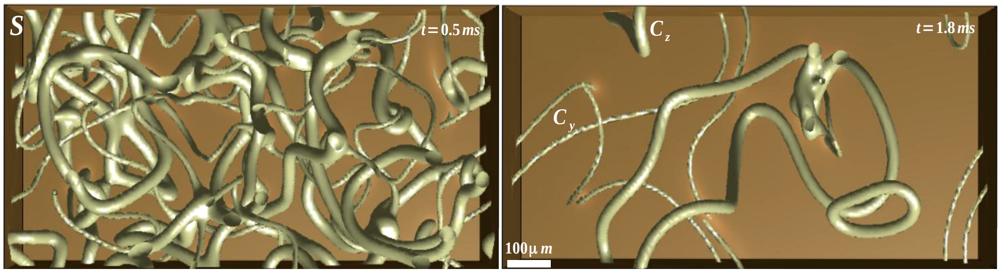

Supplementary Movie S2. Disclination kinetics in a coarsening athermal biaxial NLC. Similar to Supplementary Movie S1, this animation displays of the evolution of S & B_2 for a thermotropic biaxial NLC, subjected to a temperature quench from an isotropic phase deep into the biaxial phase. Colourbars and isovalues corresponding to the isosurfaces of disclinations are indicated in figure 1 in the main article. As $\{S, B_2\}$ grows in the media towards the saturation value $\{S_{beq}, (B_2)_{beq}\}$, isotropic domain coarsening leads to the nucleation of π solitons of C_y and C_z class at around $0.5ms$ (left panel), that intercommute only within the respective class to form contractile loops to squeeze (right panel). In B_2, strings of similar width for different isovalues as mentioned in figure 1 is coloured to distinguish. As already noted that devoid of chirality, the nonabelian disclinations of different class do not entangle but pass through each other.

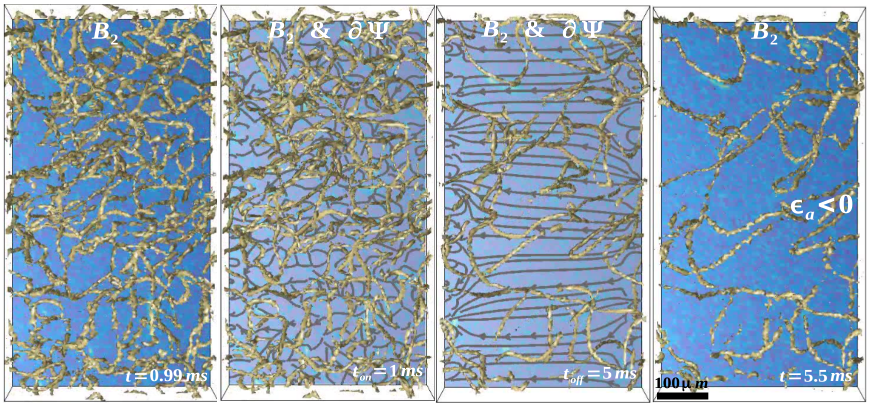

Supplementary Movie S3. Nonuniform electric flux lines and temporal dilation of disclination kinetics in a coarsening thermal uniaxial NLC. The animation sketches the evolution of $(S, \boldsymbol{\partial}\Psi)$ & $(B_2, \boldsymbol{\partial}\Psi)$ for a thermal uniaxial NLC coarsening following a temperature quench from an isotropic state, subjected to the onset of an electric field at $t_{on} = 1ms$ and cessation at $t_{off} = 5ms$ for $\varepsilon_a < 0$. Similar response for $\varepsilon_a > 0$ is not shown for brevity. Note that in the second panel after t_{on}, electric field drives the fluctuating nematic media to attain a nematic phase with $S > S_{ueq}$, however, the line defect kinetics is significantly reduced during the interval $[t_{on}, t_{off}]$. Note that disclinations are thinner after application of field and regains its equilibrium thickness after cessation of the field.

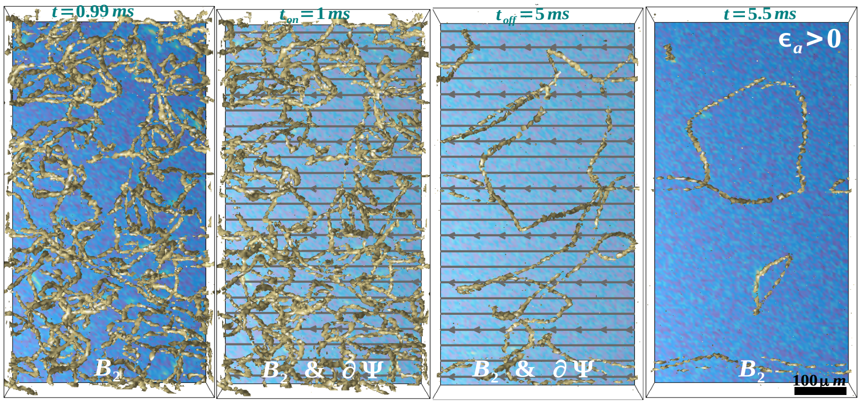

Supplementary Movie S4. Application of a uniform electric field, leading to deficient slowing down of the disclination kinetics in a coarsening thermal uniaxial NLC. The animation sketches the evolution of $(S, \boldsymbol{\partial}\Psi)$ & $(B_2, \boldsymbol{\partial}\Psi)$ for a thermal uniaxial NLC coarsening following a temperature quench from an isotropic state, subjected to the onset of an electric field at $t_{on} = 1ms$ and cessation at $t_{off} = 5ms$ for $\varepsilon_a > 0$. Similar response for $\varepsilon_a < 0$ is not shown for brevity. When compared to Supplementary Movie S3, the temporal dilation of disclination network in the presence of electric field is not substantial.

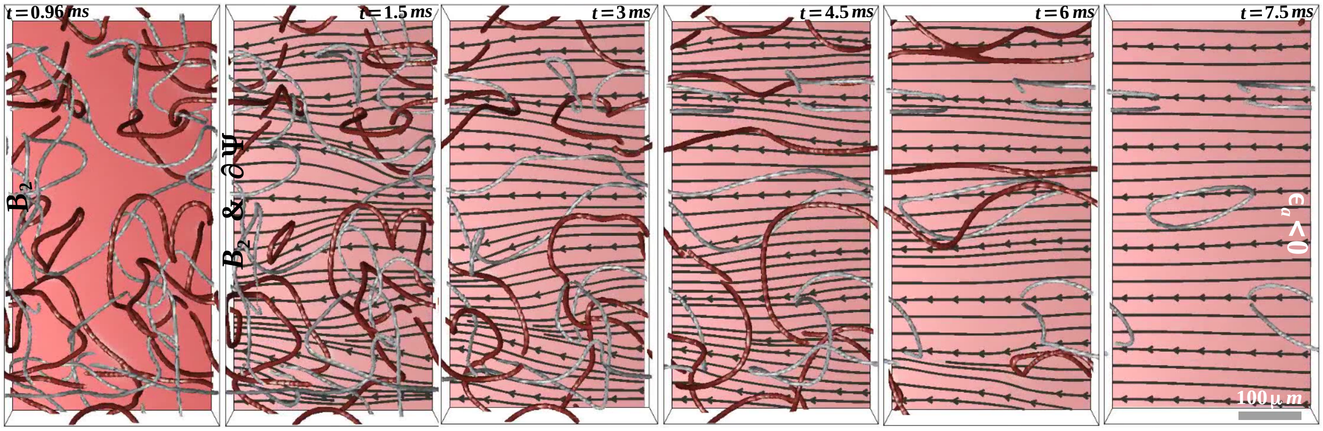

Supplementary Movie S5. Nonuniform electric flux lines, temporal dilation of disclination kinetics and selection of C_z class in a coarsening thermal biaxial NLC with $\boldsymbol{\varepsilon_a} < \mathbf{0}$. The animation sequentially portrays evolution of $(S, \boldsymbol{\partial}\Psi)$ & $(B_2, \boldsymbol{\partial}\Psi)$ for a coarsening thermal biaxial NLC following a quench from an isotropic state, subjected to the onset of an electric force at $t_{on} = 1ms$. Note that unlike Supplementary Movie S3, we do not switch off the field ($t_{off} \to \infty$). After t_{on}, the electric field drives the isotropic media to attain a biaxial nematic phase with $S > S_{beq}$, however, the disclination kinetics is sufficiently reduced. The disclinations are thinner after application of electric field. The kinetic pathway of $C_{y,z}$ become asymmetric and as a result, the C_y class is long-lived. Selection of C_y class is also seen in the solution of equation 6 in the main article (not shown). We have also noted that the equilibrium dynamics is regained after cessation of field (not shown).

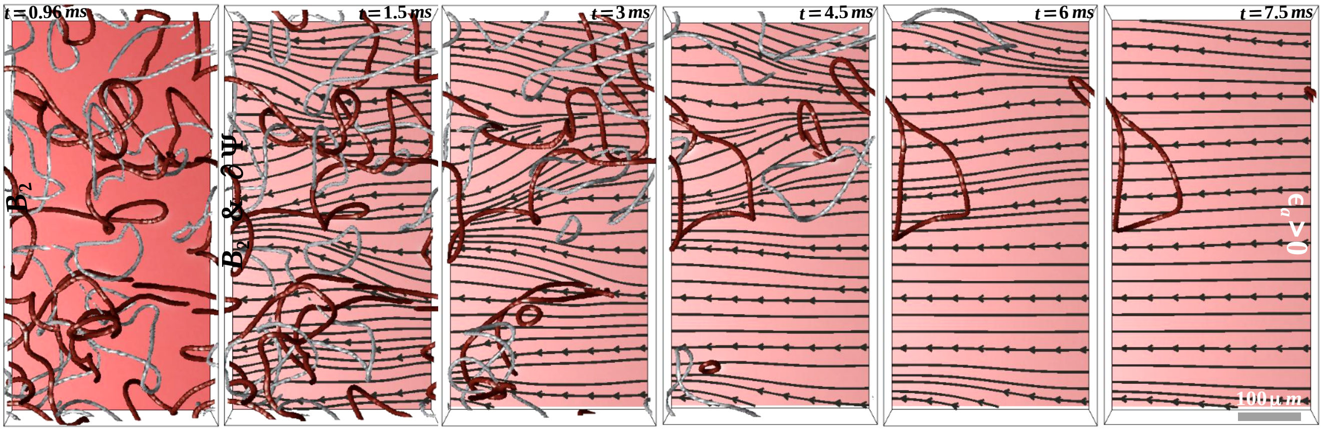

Supplementary Movie S6. Nonuniform electric flux lines, temporal dilation of disclination kinetics and selection of C_y class in a coarsening thermal biaxial NLC with $\boldsymbol{\varepsilon_a} > \mathbf{0}$. Instead of Supplementary Movie S5, here we find that C_y class is long lived.
